# Poly[[μ-aqua-bis­(μ-4,4′-bipyridine-κ^2^ *N*:*N*′)bis­(μ-3-hy­droxy­adamantane-1-carboxyl­ato-κ^2^ *O*:*O*′)bis­(3-hy­droxy­adamantane-1-carboxyl­ato-κ*O*)dicobalt(II)] hepta­hydrate]

**DOI:** 10.1107/S1600536811012475

**Published:** 2011-04-07

**Authors:** Jin-Bei Shen, Xiao-Yong Wu, Xiao-Ju Chen, Guo-Liang Zhao

**Affiliations:** a College of Chemistry and Life Science, Zhejiang Normal University, Jinhua 321004, Zhejiang, People’s Republic of China; b Zhejiang Normal University Xingzhi College, Jinhua, Zhejiang 321004, People’s Republic of China

## Abstract

The title coordination compound, {[Co(C_11_H_15_O_3_)_4_(C_10_H_8_N_2_)_2_(H_2_O)]·7H_2_O}_*n*_, consists of a pair of Co^II^ atoms, four 3-hy­droxy­adamantane-1-carboxyl­ate anions (*L*), one water mol­ecule, two bridging 4,4′-bipyridine (4,4′-bpy) ligands and seven uncoordinated water mol­ecules. Both of the Co^II^ ions are coordinated in a distorted octa­hedral geometry. Four *L* ligands bind to each pair of Co^II^ atoms in a plane, two of which bridge the two Co^II^ atoms as bidentate groups while the other two coordinate to a single Co^II^ atom in a monodentate mode. Two half-mol­ecules of 4,4′-bipyridine coordinate the Co^II^ atoms from the upside and underside. The packing features extensice O—H⋯O hydrogen bonding.

## Related literature

For a related nickel complex, see: Hu *et al.* (2011 ▶). For other complexes based on adamantane-1-carboxylic acid, see: Milios *et al.* (2007 ▶); Korlyukov *et al.* (2008 ▶); Zhu *et al.* (2005 ▶).
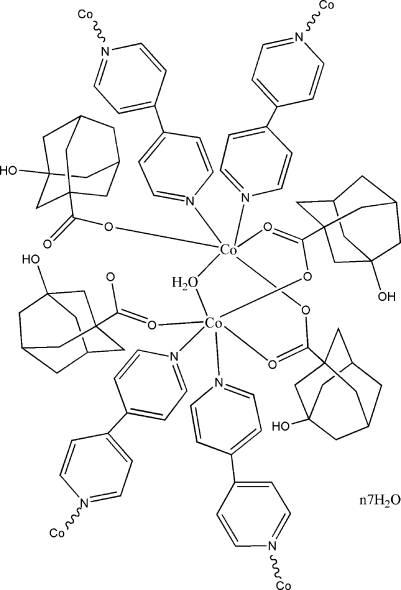


## Experimental

### 

#### Crystal data

[Co(C_11_H_15_O_3_)_4_(C_10_H_8_N_2_)_2_(H_2_O)]·7H_2_O
*M* *_r_* = 1355.28
Monoclinic, 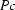

*a* = 12.0201 (3) Å
*b* = 20.7463 (5) Å
*c* = 17.6353 (3) Å
β = 132.806 (1)°
*V* = 3226.45 (12) Å^3^
*Z* = 2
Mo *K*α radiation
μ = 0.59 mm^−1^
*T* = 296 K
0.20 × 0.13 × 0.04 mm

#### Data collection

Bruker APEXII area-detector diffractometer
Absorption correction: multi-scan (*SADABS*; Sheldrick, 1996 ▶) *T*_min_ = 0.909, *T*_max_ = 0.979
42213 measured reflections
11001 independent reflections
9720 reflections with *I* > 2σ(*I*)
*R*_int_ = 0.050

#### Refinement

*R*[*F* ^2^ > 2σ(*F* ^2^)] = 0.035
*wR*(*F* ^2^) = 0.083
*S* = 1.03
11001 reflections
797 parameters
27 restraints
H-atom parameters constrained
Δρ_max_ = 0.33 e Å^−3^
Δρ_min_ = −0.34 e Å^−3^
Absolute structure: Flack (1983 ▶), 5293 Friedel pairs
Flack parameter: −0.003 (9)

### 

Data collection: *APEX2* (Bruker, 2006 ▶); cell refinement: *SAINT* (Bruker, 2006 ▶); data reduction: *SAINT*; program(s) used to solve structure: *SHELXS97* (Sheldrick, 2008 ▶); program(s) used to refine structure: *SHELXL97* (Sheldrick, 2008 ▶); molecular graphics: *SHELXTL* (Sheldrick, 2008 ▶); software used to prepare material for publication: *SHELXL97*.

## Supplementary Material

Crystal structure: contains datablocks I, global. DOI: 10.1107/S1600536811012475/om2417sup1.cif

Structure factors: contains datablocks I. DOI: 10.1107/S1600536811012475/om2417Isup2.hkl

Additional supplementary materials:  crystallographic information; 3D view; checkCIF report

## Figures and Tables

**Table 1:** Selected bond lengths (Å)

Co1—O7	2.019 (2)
Co1—O11	2.068 (2)
Co1—O5	2.1277 (19)
Co1—N1	2.145 (2)
Co1—O1*W*	2.1603 (18)
Co1—N4	2.161 (2)
Co2—O4	2.022 (2)
Co2—O2	2.072 (2)
Co2—O8	2.110 (2)
Co2—N2	2.154 (2)
Co2—O1*W*	2.1813 (19)
Co2—N3	2.198 (2)

**Table 2:** Hydrogen-bond geometry (Å, °)

*D*—H⋯*A*	*D*—H	H⋯*A*	*D*⋯*A*	*D*—H⋯*A*
O1*W*—H1*WA*⋯O1	0.85	1.78	2.623 (3)	168
O1*W*—H1*WA*⋯O2	0.85	2.56	2.968 (3)	111
O1*W*—H1*WB*⋯O10	0.95	1.69	2.623 (3)	164
O3*W*—H3*WA*⋯O5*W*	0.85	2.01	2.844 (5)	166
O3*W*—H3*WB*⋯O8*W*	0.85	2.12	2.973 (5)	179
O12—H12*A*⋯O4*W*	0.82	2.03	2.836 (4)	170
O6*W*—H6*WA*⋯O2*W*	0.85	2.08	2.865 (8)	154
O6—H6*C*⋯O8*W*	0.82	2.10	2.923 (4)	180
O6*W*—H6*WB*⋯O7*W*	0.85	2.08	2.920 (7)	167
O7*W*—H7*WB*⋯O5	0.85	2.07	2.794 (3)	143
O7*W*—H7*WA*⋯O11	0.85	2.12	2.908 (3)	155
O3—H3*C*⋯O6^i^	0.82	2.07	2.884 (4)	176
O9—H9*B*⋯O7*W*^ii^	0.82	2.04	2.864 (4)	178
O2*W*—H2*WA*⋯O3^iii^	0.85	2.03	2.881 (6)	179
O4*W*—H4*WA*⋯O8^iv^	0.84	2.10	2.943 (3)	176
O4*W*—H4*WB*⋯O1^iv^	0.82	2.09	2.843 (4)	152
O5*W*—H5*WA*⋯O12^v^	0.85	2.00	2.849 (4)	180
O5*W*—H5*WB*⋯O9^vi^	0.85	2.11	2.927 (4)	163
O8*W*—H8*WA*⋯O1^vii^	0.83	2.08	2.882 (4)	163
O8*W*—H8*WB*⋯O10^vii^	0.85	2.01	2.857 (4)	173
O2*W*—H2*WA*⋯O3^iii^	0.85	2.03	2.881 (6)	179

Symmetry codes: (i) 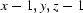; (ii) 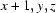; (iii) 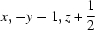; (iv) 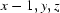; (v) 
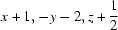
; (vi) 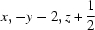; (vii) 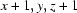.

**Fig. 1.**
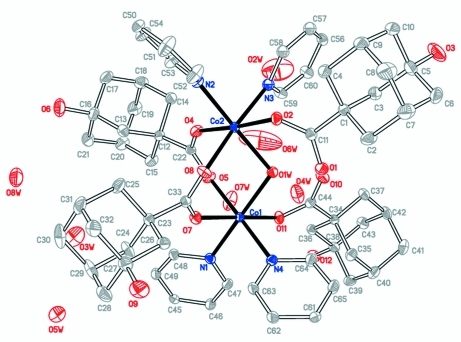
The molecular structure of the title complex, showing the atom-labeling scheme. Displacement ellipsoids are drawn at the 30% probability level.

## supplementary crystallographic information

### Comment

The fascinating structures of adamantane-1- carboxylic acid complexes coupled with their special functionality have attracted a great deal of interest (Zhu et al., 2005; Milios et al., 2007; Korlyukov et al., 2008). Recently, we reported the structure of a nickel complex with 3-hydroxyadamantane-1-carboxylic acid and 4,4'-bpy (Hu et al., 2011). As an extension of our work in this field, we describe a new Co^II^ complex.

The structure of the Co complex is shown in Fig. 1. It is constructed by a central cobalt unit, and each unit consists of a pair of Co^II^ centers, four 3- hydroxy-adamantane-1-carboxylic acid anions (L), one water molecule, two bridging 4,4'-bpy ligands and seven uncoordinated water molecules. Four L  ligands bind to each pair of Co^II^ center in a plane, two of which bridge the two Co^II^ centers as bidentate bridging ligands, while the other two coordinate to a single Co^II^ center in monodentate mode. Two half parts of 4,4' -bipyridine coordinate Co(1) and Co(2) from the upside and underside. One coordinated water molecule bridges the Co(1) and Co(2) with a similar bond length [Co(1)—O(1W) = 2.160 (3)Å, Co(2)—O(1W) = 2.181 (6)Å]. The structure demonstrates that both of the Co^II^ ions coordinate in a distorted octahedral geometry. The Co—O (from carboxylic and water oxygen) distances are all within the range 2.019 (2)-2.127 (3)Å, and the Co—N distances range from 2.145 (2)-2.198 (3)Å. The selected bond lengths and angles for the complex are listed in Table 1.

The binuclear unit is further supported by hydrogen bonding interactions involving the non-coordinated oxygen atoms of the two monodentate L ligands, one bridging water molecule and seven uncoordinated water molecules. The hydrogen bonds are listed in Table 2.

### Experimental

Reagents and solvents used were of commercially available quality and without purified before using. A mixture of 3-hydroxyadamantane-1-carboxylic acid (0.3924 g, 2 mmol), CoSO_4_^.^7H_2_O (0.2811 g, 1 mmol), 4,4'-bipyridine (0.1562 g, 1 mmol) and water (16 ml) was sealed in a 25 ml stainless steel reactor with a Teflon liner and heated at 160 K for 2 d and then cooled to room temperature over 3 d. The resulting pink crystals suitable for X-ray diffraction were obtained and collected by filtration, washed with water, and evaporated in air for one month.

### Refinement

The structure was solved by direct methods and successive Fourier difference synthesis. The H atoms bonded to C atoms were positioned geometrically and refined using a riding model [aliphatic C—H =0.96 Å (*U*_iso_(H) = 1.5*U*_eq_(C)), aromatic C—H = 0.93 Å (*U*_iso_(H) = 1.2*U*_eq_(C))]. H atoms bonded to O atoms were located in difference Fourier maps and refined with O—H distance restraints of 0.83 (2) and *U*_iso_(H) = 1.5*U*_eq_(O).

### Crystal data

**Table:**

[Co(C_11_H_15_O_3_)_4_(C_10_H_8_N_2_)_2_(H_2_O)]·7H_2_O	*F*(000) = 1436
*M**_r_* = 1355.28	*D*_x_ = 1.395 Mg m^−^^3^
Monoclinic, *P**c*	Mo *K*α radiation, λ = 0.71073 Å
Hall symbol: P -2yc	Cell parameters from 8372 reflections
*a* = 12.0201 (3) Å	θ = 1.9–25.0°
*b* = 20.7463 (5) Å	µ = 0.59 mm^−^^1^
*c* = 17.6353 (3) Å	*T* = 296 K
β = 132.806 (1)°	Block, red
*V* = 3226.45 (12) Å^3^	0.20 × 0.13 × 0.04 mm
*Z* = 2	

### Data collection

**Table:**

Bruker APEXII area-detector diffractometer	11001 independent reflections
Radiation source: fine-focus sealed tube	9720 reflections with *I* > 2σ(*I*)
graphite	*R*_int_ = 0.050
φ and ω scans	θ_max_ = 25.0°, θ_min_ = 1.9°
Absorption correction: multi-scan (*SADABS*; Sheldrick, 1996)	*h* = −14→14
*T*_min_ = 0.909, *T*_max_ = 0.979	*k* = −24→24
42213 measured reflections	*l* = −20→20

### Refinement

**Table:**

Refinement on *F*^2^	Hydrogen site location: inferred from neighbouring sites
Least-squares matrix: full	H-atom parameters constrained
*R*[*F*^2^ > 2σ(*F*^2^)] = 0.035	*w* = 1/[σ^2^(*F*_o_^2^) + (0.0422*P*)^2^ + 0.0362*P*] where *P* = (*F*_o_^2^ + 2*F*_c_^2^)/3
*wR*(*F*^2^) = 0.083	(Δ/σ)_max_ = 0.001
*S* = 1.03	Δρ_max_ = 0.33 e Å^−^^3^
11001 reflections	Δρ_min_ = −0.34 e Å^−^^3^
797 parameters	Extinction correction: *SHELXL97* (Sheldrick, 2008), Fc^*^=kFc[1+0.001xFc^2^λ^3^/sin(2θ)]^-1/4^
27 restraints	Extinction coefficient: 0.0005 (2)
Primary atom site location: structure-invariant direct methods	Absolute structure: Flack (1983), 5293 Friedel pairs
Secondary atom site location: difference Fourier map	Flack parameter: −0.003 (9)

### Special details

**Table:**

Geometry. All e.s.d.'s (except the e.s.d. in the dihedral angle between two l.s. planes) are estimated using the full covariance matrix. The cell e.s.d.'s are taken into account individually in the estimation of e.s.d.'s in distances, angles and torsion angles; correlations between e.s.d.'s in cell parameters are only used when they are defined by crystal symmetry. An approximate (isotropic) treatment of cell e.s.d.'s is used for estimating e.s.d.'s involving l.s. planes.
Refinement. Refinement of *F*^2^ against ALL reflections. The weighted *R*-factor *wR* and goodness of fit *S* are based on *F*^2^, conventional *R*-factors *R* are based on *F*, with *F* set to zero for negative *F*^2^. The threshold expression of *F*^2^ > σ(*F*^2^) is used only for calculating *R*-factors(gt) *etc*. and is not relevant to the choice of reflections for refinement. *R*-factors based on *F*^2^ are statistically about twice as large as those based on *F*, and *R*- factors based on ALL data will be even larger.

### Fractional atomic coordinates and isotropic or equivalent isotropic displacement parameters (Å^2^)

**Table:**

	*x*	*y*	*z*	*U*_iso_*/*U*_eq_	
Co1	−0.18916 (3)	−0.826639 (16)	0.11759 (2)	0.01865 (10)	
Co2	−0.05072 (3)	−0.673840 (16)	0.11470 (2)	0.01938 (10)	
O1	−0.2061 (3)	−0.73886 (10)	−0.11757 (16)	0.0340 (5)	
O1W	−0.2231 (2)	−0.74854 (9)	0.02265 (14)	0.0232 (4)	
H1WA	−0.2192	−0.7514	−0.0237	0.035*	
H1WB	−0.3310	−0.7452	−0.0271	0.035*	
O2	−0.0600 (2)	−0.66001 (9)	−0.00587 (15)	0.0289 (5)	
O3	−0.5154 (3)	−0.55673 (13)	−0.4035 (2)	0.0745 (10)	
H3C	−0.5666	−0.5795	−0.3991	0.112*	
O4	−0.0446 (3)	−0.68089 (9)	0.23185 (16)	0.0301 (5)	
O5	−0.1817 (2)	−0.76186 (9)	0.21450 (15)	0.0279 (5)	
O6	0.3178 (3)	−0.64086 (13)	0.6193 (2)	0.0615 (7)	
H6C	0.3607	−0.6744	0.6511	0.092*	
O7	0.0395 (2)	−0.82268 (9)	0.21983 (16)	0.0285 (5)	
O8	0.1188 (2)	−0.74459 (9)	0.17905 (16)	0.0305 (5)	
O9	0.5482 (3)	−0.90783 (12)	0.2854 (2)	0.0515 (7)	
H9B	0.5553	−0.8806	0.2552	0.077*	
O10	−0.5180 (2)	−0.75975 (10)	−0.10005 (17)	0.0389 (6)	
O11	−0.4217 (2)	−0.83275 (9)	0.02313 (15)	0.0277 (5)	
O12	−0.8771 (3)	−0.92293 (10)	−0.04898 (17)	0.0371 (5)	
H12A	−0.8920	−0.8923	−0.0273	0.056*	
N1	−0.1695 (3)	−0.90216 (11)	0.20915 (18)	0.0246 (5)	
N2	0.1235 (3)	−0.60113 (12)	0.19921 (18)	0.0266 (6)	
C1	−0.1645 (4)	−0.64664 (14)	−0.1779 (2)	0.0261 (6)	
C2	−0.1469 (5)	−0.68941 (16)	−0.2403 (3)	0.0413 (9)	
H2A	−0.0437	−0.7055	−0.1955	0.050*	
H2B	−0.2148	−0.7261	−0.2682	0.050*	
C3	−0.3278 (4)	−0.62070 (15)	−0.2504 (2)	0.0361 (8)	
H3A	−0.3981	−0.6564	−0.2782	0.043*	
H3B	−0.3396	−0.5933	−0.2117	0.043*	
C4	−0.0565 (4)	−0.58952 (15)	−0.1341 (2)	0.0323 (7)	
H4A	0.0472	−0.6049	−0.0889	0.039*	
H4B	−0.0659	−0.5625	−0.0938	0.039*	
C5	−0.3645 (4)	−0.58258 (16)	−0.3382 (3)	0.0453 (9)	
C6	−0.3459 (6)	−0.62580 (18)	−0.3992 (3)	0.0612 (13)	
H6A	−0.4150	−0.6620	−0.4278	0.073*	
H6B	−0.3701	−0.6017	−0.4559	0.073*	
C7	−0.1839 (6)	−0.65009 (18)	−0.3284 (3)	0.0547 (11)	
H7	−0.1722	−0.6774	−0.3680	0.066*	
C8	−0.0745 (5)	−0.59261 (18)	−0.2831 (3)	0.0564 (11)	
H8A	0.0293	−0.6080	−0.2378	0.068*	
H8B	−0.0955	−0.5680	−0.3384	0.068*	
C9	−0.0944 (4)	−0.55008 (16)	−0.2228 (3)	0.0393 (8)	
H9A	−0.0251	−0.5133	−0.1942	0.047*	
C10	−0.2565 (5)	−0.52564 (16)	−0.2942 (3)	0.0471 (10)	
H10A	−0.2794	−0.5008	−0.3500	0.057*	
H10B	−0.2687	−0.4978	−0.2561	0.057*	
C11	−0.1398 (3)	−0.68428 (14)	−0.0934 (2)	0.0261 (7)	
C12	−0.0568 (3)	−0.68948 (13)	0.3591 (2)	0.0246 (6)	
C13	0.1157 (4)	−0.68060 (15)	0.4456 (2)	0.0331 (7)	
H13A	0.1655	−0.7220	0.4630	0.040*	
H13B	0.1511	−0.6525	0.4218	0.040*	
C14	−0.1329 (4)	−0.62352 (14)	0.3347 (2)	0.0326 (7)	
H14A	−0.1015	−0.5946	0.3090	0.039*	
H14B	−0.2421	−0.6284	0.2812	0.039*	
C15	−0.1095 (4)	−0.73446 (15)	0.3991 (3)	0.0358 (8)	
H15A	−0.2186	−0.7401	0.3461	0.043*	
H15B	−0.0624	−0.7764	0.4154	0.043*	
C16	0.1570 (4)	−0.65150 (17)	0.5415 (2)	0.0392 (8)	
C17	0.0811 (5)	−0.58674 (16)	0.5150 (3)	0.0463 (9)	
H17A	0.1153	−0.5580	0.4910	0.056*	
H17B	0.1086	−0.5679	0.5762	0.056*	
C18	−0.0896 (4)	−0.59461 (16)	0.4315 (3)	0.0417 (8)	
H18A	−0.1384	−0.5525	0.4148	0.050*	
C19	−0.1445 (5)	−0.64023 (18)	0.4689 (3)	0.0487 (10)	
H19A	−0.1213	−0.6220	0.5289	0.058*	
H19B	−0.2535	−0.6459	0.4148	0.058*	
C20	−0.0654 (4)	−0.70500 (17)	0.4965 (3)	0.0422 (9)	
H20	−0.0985	−0.7339	0.5219	0.051*	
C21	0.1039 (5)	−0.69680 (18)	0.5792 (3)	0.0466 (9)	
H21A	0.1530	−0.7384	0.5962	0.056*	
H21B	0.1316	−0.6793	0.6412	0.056*	
C22	−0.0982 (3)	−0.71336 (13)	0.2607 (2)	0.0236 (6)	
C23	0.3031 (3)	−0.81885 (13)	0.3131 (2)	0.0248 (6)	
C24	0.3128 (4)	−0.87058 (18)	0.3799 (3)	0.0400 (8)	
H24A	0.2862	−0.8518	0.4162	0.048*	
H24B	0.2417	−0.9051	0.3362	0.048*	
C25	0.4151 (4)	−0.76443 (16)	0.3829 (3)	0.0400 (8)	
H25A	0.3882	−0.7442	0.4182	0.048*	
H25B	0.4111	−0.7320	0.3415	0.048*	
C26	0.3478 (3)	−0.85009 (15)	0.2583 (2)	0.0292 (7)	
H26A	0.2772	−0.8845	0.2138	0.035*	
H26B	0.3420	−0.8183	0.2155	0.035*	
C27	0.5082 (4)	−0.87722 (16)	0.3367 (3)	0.0362 (8)	
C28	0.5163 (4)	−0.92747 (17)	0.4023 (3)	0.0472 (9)	
H28A	0.4474	−0.9626	0.3589	0.057*	
H28B	0.6183	−0.9449	0.4523	0.057*	
C29	0.4742 (4)	−0.8976 (2)	0.4580 (3)	0.0520 (9)	
H29	0.4797	−0.9306	0.5002	0.062*	
C30	0.5839 (5)	−0.8432 (2)	0.5278 (3)	0.0640 (11)	
H30A	0.5570	−0.8242	0.5637	0.077*	
H30B	0.6862	−0.8601	0.5790	0.077*	
C31	0.5765 (4)	−0.79274 (18)	0.4627 (3)	0.0478 (9)	
H31	0.6481	−0.7581	0.5078	0.057*	
C32	0.6190 (4)	−0.82253 (16)	0.4058 (3)	0.0422 (9)	
H32A	0.6142	−0.7900	0.3642	0.051*	
H32B	0.7218	−0.8391	0.4554	0.051*	
C33	0.1416 (3)	−0.79277 (13)	0.2320 (2)	0.0241 (6)	
C34	−0.6859 (3)	−0.84038 (13)	−0.1254 (2)	0.0235 (6)	
C35	−0.6842 (4)	−0.89964 (15)	−0.1771 (2)	0.0324 (7)	
H35A	−0.6704	−0.8860	−0.2229	0.039*	
H35B	−0.6003	−0.9276	−0.1247	0.039*	
C36	−0.7108 (3)	−0.86335 (14)	−0.0550 (2)	0.0257 (6)	
H36A	−0.6275	−0.8910	−0.0014	0.031*	
H36B	−0.7123	−0.8264	−0.0220	0.031*	
C37	−0.8186 (3)	−0.79611 (15)	−0.2094 (2)	0.0309 (7)	
H37A	−0.8209	−0.7587	−0.1774	0.037*	
H37B	−0.8050	−0.7812	−0.2547	0.037*	
C38	−0.8593 (3)	−0.90004 (14)	−0.1166 (2)	0.0278 (7)	
C39	−0.8548 (4)	−0.95843 (15)	−0.1668 (3)	0.0369 (8)	
H39A	−0.7716	−0.9865	−0.1138	0.044*	
H39B	−0.9485	−0.9826	−0.2056	0.044*	
C40	−0.8339 (4)	−0.93638 (16)	−0.2387 (2)	0.0364 (8)	
H40	−0.8317	−0.9742	−0.2710	0.044*	
C41	−0.9649 (4)	−0.89244 (17)	−0.3230 (2)	0.0427 (9)	
H41A	−1.0601	−0.9157	−0.3634	0.051*	
H41B	−0.9515	−0.8788	−0.3689	0.051*	
C42	−0.9686 (4)	−0.83328 (16)	−0.2722 (3)	0.0362 (8)	
H42	−1.0530	−0.8051	−0.3258	0.043*	
C43	−0.9907 (4)	−0.85626 (16)	−0.2007 (2)	0.0348 (8)	
H43A	−0.9953	−0.8194	−0.1691	0.042*	
H43B	−1.0856	−0.8796	−0.2404	0.042*	
C44	−0.5320 (3)	−0.80707 (14)	−0.0634 (2)	0.0249 (6)	
C45	−0.1753 (3)	−0.98457 (13)	0.3330 (2)	0.0235 (6)	
C46	−0.2750 (4)	−0.99210 (14)	0.2260 (2)	0.0300 (7)	
H46	−0.3459	−1.0253	0.1937	0.036*	
C47	−0.2686 (4)	−0.95060 (14)	0.1684 (2)	0.0297 (7)	
H47	−0.3370	−0.9566	0.0972	0.036*	
C48	−0.0689 (3)	−0.89626 (14)	0.3128 (2)	0.0261 (7)	
H48	0.0045	−0.8642	0.3435	0.031*	
C49	−0.0695 (4)	−0.93522 (14)	0.3752 (2)	0.0289 (7)	
H49	0.0014	−0.9286	0.4463	0.035*	
C50	0.3717 (3)	−0.51750 (14)	0.3291 (2)	0.0284 (7)	
C51	0.3417 (4)	−0.55068 (19)	0.2489 (3)	0.0502 (10)	
H51	0.4045	−0.5450	0.2362	0.060*	
C52	0.2213 (4)	−0.59158 (18)	0.1883 (3)	0.0462 (10)	
H52	0.2068	−0.6140	0.1366	0.055*	
C53	0.1483 (4)	−0.56755 (15)	0.2740 (2)	0.0327 (7)	
H53	0.0808	−0.5727	0.2828	0.039*	
C54	0.2680 (4)	−0.52567 (16)	0.3389 (3)	0.0366 (8)	
H54	0.2789	−0.5030	0.3891	0.044*	
O2W	−0.6463 (7)	−0.5698 (2)	0.0516 (5)	0.164 (2)	
H2WA	−0.6071	−0.5326	0.0650	0.246*	
H2WB	−0.7227	−0.5627	0.0445	0.246*	
O3W	0.2700 (5)	−0.8634 (2)	0.5795 (3)	0.1280 (17)	
H3WA	0.2757	−0.9038	0.5895	0.192*	
H3WB	0.3270	−0.8337	0.6229	0.192*	
O4W	−0.9266 (4)	−0.80714 (16)	0.0099 (2)	0.0814 (11)	
H4WA	−0.9120	−0.7873	0.0579	0.122*	
H4WB	−1.0151	−0.7979	−0.0406	0.122*	
O5W	0.3392 (4)	−0.99676 (14)	0.6252 (3)	0.0951 (12)	
H5WA	0.2746	−1.0206	0.5731	0.143*	
H5WB	0.3925	−1.0296	0.6606	0.143*	
O6W	−0.6353 (11)	−0.7012 (3)	0.1063 (9)	0.269 (5)	
H6WA	−0.6073	−0.6639	0.1051	0.403*	
H6WB	−0.5697	−0.7312	0.1379	0.403*	
O7W	−0.4278 (3)	−0.80967 (14)	0.1829 (2)	0.0584 (7)	
H7WA	−0.4551	−0.8186	0.1252	0.088*	
H7WB	−0.3455	−0.7887	0.2167	0.088*	
O8W	0.4710 (3)	−0.76040 (14)	0.7329 (2)	0.0656 (8)	
H8WA	0.5634	−0.7586	0.7672	0.098*	
H8WB	0.4667	−0.7583	0.7793	0.098*	
C62	−0.0705 (4)	−0.97459 (15)	0.0048 (2)	0.0336 (8)	
H62	0.0118	−1.0023	0.0379	0.040*	
C65	−0.2990 (4)	−0.93054 (16)	−0.1448 (2)	0.0354 (8)	
H65	−0.3772	−0.9277	−0.2162	0.042*	
N4	−0.1940 (3)	−0.89317 (11)	0.02111 (18)	0.0241 (5)	
C63	−0.0812 (4)	−0.93489 (14)	0.0621 (2)	0.0317 (7)	
H63	−0.0052	−0.9373	0.1338	0.038*	
C64	−0.3012 (4)	−0.89177 (15)	−0.0822 (2)	0.0322 (7)	
H64	−0.3815	−0.8631	−0.1136	0.039*	
C61	−0.1825 (3)	−0.97326 (13)	−0.1024 (2)	0.0232 (6)	
C56	−0.4934 (3)	−0.52461 (14)	−0.0996 (2)	0.0283 (7)	
C57	−0.3573 (4)	−0.51075 (15)	−0.0728 (2)	0.0332 (7)*	
H57	−0.3497	−0.4745	−0.1001	0.040*	
C58	−0.2339 (4)	−0.55035 (14)	−0.0060 (2)	0.0310 (7)*	
H58	−0.1444	−0.5400	0.0103	0.037*	
C59	−0.3642 (4)	−0.61572 (14)	0.0135 (2)	0.0310 (7)	
H59	−0.3668	−0.6512	0.0445	0.037*	
N3	−0.2360 (3)	−0.60329 (12)	0.03679 (19)	0.0276 (6)*	
C60	−0.4943 (4)	−0.57922 (15)	−0.0539 (3)	0.0337 (7)	
H60	−0.5820	−0.5910	−0.0688	0.040*	

### Atomic displacement parameters (Å^2^)

**Table:**

	*U*^11^	*U*^22^	*U*^33^	*U*^12^	*U*^13^	*U*^23^
Co1	0.0187 (2)	0.01868 (19)	0.01874 (19)	−0.00078 (17)	0.01282 (17)	−0.00001 (17)
Co2	0.0197 (2)	0.0191 (2)	0.01956 (19)	−0.00031 (17)	0.01342 (18)	0.00082 (16)
O1	0.0428 (14)	0.0279 (11)	0.0310 (12)	−0.0062 (10)	0.0249 (12)	0.0014 (9)
O1W	0.0227 (11)	0.0233 (10)	0.0239 (11)	−0.0015 (8)	0.0159 (10)	0.0009 (8)
O2	0.0312 (12)	0.0312 (11)	0.0219 (11)	−0.0045 (9)	0.0172 (10)	−0.0002 (9)
O3	0.0470 (18)	0.0617 (18)	0.0592 (18)	0.0066 (14)	0.0140 (15)	0.0329 (15)
O4	0.0416 (13)	0.0282 (11)	0.0295 (11)	−0.0097 (10)	0.0277 (11)	−0.0039 (9)
O5	0.0325 (12)	0.0257 (11)	0.0336 (12)	−0.0083 (9)	0.0257 (11)	−0.0099 (9)
O6	0.0421 (15)	0.0726 (18)	0.0327 (13)	−0.0114 (15)	0.0107 (12)	0.0036 (14)
O7	0.0195 (11)	0.0328 (11)	0.0307 (11)	−0.0002 (9)	0.0160 (10)	0.0032 (9)
O8	0.0308 (12)	0.0269 (11)	0.0384 (12)	0.0089 (9)	0.0254 (11)	0.0121 (9)
O9	0.0438 (16)	0.0522 (15)	0.0649 (17)	0.0106 (12)	0.0394 (15)	−0.0018 (13)
O10	0.0246 (12)	0.0369 (12)	0.0409 (13)	−0.0022 (10)	0.0167 (11)	0.0126 (10)
O11	0.0169 (11)	0.0359 (12)	0.0229 (11)	−0.0037 (9)	0.0106 (10)	0.0019 (9)
O12	0.0353 (13)	0.0365 (12)	0.0464 (14)	−0.0009 (10)	0.0305 (12)	0.0063 (10)
N1	0.0266 (14)	0.0243 (13)	0.0238 (13)	0.0006 (11)	0.0174 (12)	0.0034 (10)
N2	0.0252 (14)	0.0268 (13)	0.0275 (13)	−0.0032 (11)	0.0177 (12)	−0.0040 (10)
C1	0.0342 (18)	0.0242 (15)	0.0242 (15)	−0.0015 (13)	0.0216 (15)	0.0020 (12)
C2	0.067 (3)	0.0292 (17)	0.044 (2)	−0.0056 (16)	0.045 (2)	−0.0056 (15)
C3	0.036 (2)	0.0315 (17)	0.0324 (18)	−0.0052 (14)	0.0202 (17)	0.0011 (14)
C4	0.0381 (19)	0.0305 (16)	0.0292 (16)	−0.0084 (14)	0.0232 (16)	−0.0017 (13)
C5	0.043 (2)	0.0400 (19)	0.0305 (18)	−0.0030 (16)	0.0158 (18)	0.0095 (15)
C6	0.094 (4)	0.045 (2)	0.0278 (19)	−0.028 (2)	0.035 (2)	−0.0082 (17)
C7	0.098 (4)	0.039 (2)	0.053 (2)	−0.010 (2)	0.062 (3)	−0.0080 (18)
C8	0.089 (3)	0.048 (2)	0.065 (3)	−0.013 (2)	0.065 (3)	0.000 (2)
C9	0.058 (2)	0.0282 (17)	0.040 (2)	−0.0135 (16)	0.036 (2)	−0.0022 (14)
C10	0.067 (3)	0.0289 (18)	0.0375 (19)	−0.0088 (17)	0.032 (2)	0.0000 (15)
C11	0.0248 (16)	0.0275 (16)	0.0250 (16)	0.0033 (13)	0.0165 (14)	0.0028 (13)
C12	0.0302 (17)	0.0249 (15)	0.0242 (15)	−0.0014 (12)	0.0206 (14)	−0.0010 (12)
C13	0.0345 (19)	0.0339 (17)	0.0313 (17)	0.0018 (14)	0.0226 (16)	0.0043 (14)
C14	0.0343 (19)	0.0327 (16)	0.0298 (16)	0.0027 (14)	0.0214 (16)	−0.0004 (14)
C15	0.049 (2)	0.0353 (17)	0.0368 (18)	−0.0120 (16)	0.0348 (18)	−0.0041 (14)
C16	0.037 (2)	0.0433 (19)	0.0268 (17)	−0.0096 (16)	0.0172 (17)	−0.0050 (14)
C17	0.063 (3)	0.0385 (19)	0.0333 (19)	−0.0163 (18)	0.031 (2)	−0.0105 (15)
C18	0.058 (2)	0.0347 (18)	0.044 (2)	0.0024 (17)	0.039 (2)	−0.0033 (16)
C19	0.063 (3)	0.055 (2)	0.047 (2)	−0.002 (2)	0.044 (2)	−0.0078 (18)
C20	0.062 (3)	0.0419 (19)	0.040 (2)	−0.0137 (18)	0.041 (2)	−0.0054 (16)
C21	0.063 (3)	0.047 (2)	0.0292 (18)	−0.0060 (19)	0.031 (2)	0.0050 (16)
C22	0.0239 (16)	0.0213 (15)	0.0247 (15)	0.0039 (12)	0.0161 (14)	0.0024 (12)
C23	0.0180 (15)	0.0272 (15)	0.0254 (15)	−0.0021 (12)	0.0132 (14)	0.0010 (12)
C24	0.0225 (18)	0.053 (2)	0.0374 (19)	0.0117 (15)	0.0174 (16)	0.0223 (16)
C25	0.0272 (18)	0.0415 (19)	0.0377 (19)	−0.0077 (15)	0.0167 (16)	−0.0140 (15)
C26	0.0225 (16)	0.0258 (15)	0.0354 (17)	0.0011 (13)	0.0181 (15)	0.0008 (13)
C27	0.0261 (18)	0.0367 (18)	0.0412 (19)	0.0044 (14)	0.0210 (16)	0.0006 (15)
C28	0.0268 (19)	0.043 (2)	0.053 (2)	0.0088 (16)	0.0199 (18)	0.0149 (17)
C29	0.031 (2)	0.073 (2)	0.041 (2)	0.0169 (15)	0.0205 (17)	0.0334 (16)
C30	0.028 (2)	0.113 (3)	0.029 (2)	0.0103 (18)	0.0104 (18)	0.0049 (19)
C31	0.0221 (18)	0.058 (2)	0.038 (2)	−0.0062 (17)	0.0102 (17)	−0.0157 (18)
C32	0.0204 (18)	0.047 (2)	0.050 (2)	0.0020 (15)	0.0205 (18)	0.0021 (17)
C33	0.0251 (16)	0.0262 (16)	0.0230 (15)	0.0024 (13)	0.0170 (14)	−0.0006 (12)
C34	0.0164 (15)	0.0244 (15)	0.0234 (15)	0.0025 (12)	0.0110 (14)	0.0015 (12)
C35	0.0280 (18)	0.0361 (17)	0.0325 (17)	−0.0032 (14)	0.0203 (16)	−0.0045 (14)
C36	0.0212 (15)	0.0255 (15)	0.0268 (15)	0.0012 (12)	0.0149 (14)	0.0022 (12)
C37	0.0239 (17)	0.0332 (17)	0.0303 (16)	0.0055 (13)	0.0164 (15)	0.0093 (14)
C38	0.0249 (17)	0.0269 (15)	0.0310 (16)	−0.0019 (13)	0.0188 (15)	0.0022 (13)
C39	0.0311 (19)	0.0265 (17)	0.043 (2)	−0.0076 (14)	0.0209 (17)	−0.0043 (14)
C40	0.0283 (18)	0.0391 (19)	0.0359 (18)	−0.0050 (15)	0.0194 (17)	−0.0127 (15)
C41	0.033 (2)	0.057 (2)	0.0266 (17)	−0.0107 (17)	0.0158 (17)	−0.0088 (16)
C42	0.0223 (17)	0.043 (2)	0.0288 (17)	0.0050 (14)	0.0118 (15)	0.0093 (14)
C43	0.0208 (17)	0.0376 (18)	0.0395 (19)	0.0018 (14)	0.0179 (16)	0.0049 (15)
C44	0.0252 (17)	0.0250 (15)	0.0275 (16)	−0.0011 (12)	0.0191 (15)	−0.0036 (12)
C45	0.0248 (16)	0.0217 (14)	0.0258 (15)	0.0042 (12)	0.0179 (14)	0.0038 (12)
C46	0.0323 (18)	0.0286 (16)	0.0259 (16)	−0.0083 (14)	0.0185 (15)	−0.0005 (13)
C47	0.0357 (19)	0.0273 (16)	0.0245 (15)	−0.0058 (14)	0.0198 (15)	−0.0037 (13)
C48	0.0232 (17)	0.0274 (16)	0.0231 (15)	−0.0038 (12)	0.0139 (14)	0.0012 (12)
C49	0.0264 (17)	0.0314 (16)	0.0227 (15)	−0.0022 (13)	0.0142 (14)	0.0013 (13)
C50	0.0270 (17)	0.0271 (16)	0.0301 (16)	−0.0085 (13)	0.0190 (15)	−0.0067 (13)
C51	0.045 (2)	0.074 (3)	0.052 (2)	−0.031 (2)	0.041 (2)	−0.028 (2)
C52	0.045 (2)	0.063 (2)	0.048 (2)	−0.0255 (19)	0.039 (2)	−0.0270 (18)
C53	0.0327 (19)	0.0345 (17)	0.0397 (18)	−0.0089 (14)	0.0281 (17)	−0.0109 (14)
C54	0.041 (2)	0.0356 (18)	0.0417 (19)	−0.0123 (15)	0.0311 (18)	−0.0161 (15)
O2W	0.208 (6)	0.139 (4)	0.255 (7)	−0.038 (4)	0.201 (6)	−0.017 (4)
O3W	0.132 (4)	0.086 (3)	0.088 (3)	−0.018 (3)	0.043 (3)	−0.019 (2)
O4W	0.087 (3)	0.105 (2)	0.0473 (17)	0.057 (2)	0.0440 (19)	0.0155 (16)
O5W	0.087 (3)	0.0534 (18)	0.070 (2)	−0.0074 (18)	0.024 (2)	−0.0078 (17)
O6W	0.345 (12)	0.100 (4)	0.489 (14)	0.071 (6)	0.334 (11)	0.063 (7)
O7W	0.0445 (16)	0.093 (2)	0.0468 (15)	−0.0228 (15)	0.0346 (14)	−0.0213 (15)
O8W	0.0486 (17)	0.0765 (19)	0.0436 (15)	−0.0128 (15)	0.0202 (14)	0.0047 (14)
C62	0.0373 (19)	0.0310 (16)	0.0268 (16)	0.0118 (14)	0.0195 (16)	0.0019 (13)
C65	0.0325 (19)	0.0429 (19)	0.0243 (16)	0.0039 (15)	0.0167 (15)	−0.0065 (14)
N4	0.0275 (14)	0.0235 (12)	0.0235 (13)	0.0001 (10)	0.0181 (12)	−0.0011 (10)
C63	0.0352 (19)	0.0324 (17)	0.0213 (15)	0.0094 (14)	0.0168 (15)	0.0023 (13)
C64	0.0290 (18)	0.0368 (18)	0.0261 (16)	0.0053 (14)	0.0168 (15)	−0.0017 (14)
C61	0.0290 (17)	0.0210 (14)	0.0253 (16)	−0.0024 (12)	0.0208 (15)	−0.0014 (12)
C56	0.0267 (17)	0.0309 (16)	0.0265 (16)	0.0093 (13)	0.0177 (15)	0.0048 (13)
C59	0.0330 (18)	0.0267 (16)	0.0395 (18)	0.0053 (14)	0.0272 (16)	0.0080 (14)
C60	0.0278 (18)	0.0348 (18)	0.0412 (18)	0.0043 (14)	0.0245 (16)	0.0078 (15)

### Geometric parameters (Å, °)

**Table:**

Co1—O7	2.019 (2)	C26—C27	1.525 (4)
Co1—O11	2.068 (2)	C26—H26A	0.9700
Co1—O5	2.1277 (19)	C26—H26B	0.9700
Co1—N1	2.145 (2)	C27—C28	1.511 (5)
Co1—O1W	2.1603 (18)	C27—C32	1.530 (5)
Co1—N4	2.161 (2)	C28—C29	1.510 (6)
Co2—O4	2.022 (2)	C28—H28A	0.9700
Co2—O2	2.072 (2)	C28—H28B	0.9700
Co2—O8	2.110 (2)	C29—C30	1.523 (6)
Co2—N2	2.154 (2)	C29—H29	0.9800
Co2—O1W	2.1813 (19)	C30—C31	1.511 (6)
Co2—N3	2.198 (2)	C30—H30A	0.9700
O1—C11	1.280 (3)	C30—H30B	0.9700
O1W—H1WA	0.8525	C31—C32	1.532 (5)
O1W—H1WB	0.9546	C31—H31	0.9800
O2—C11	1.242 (3)	C32—H32A	0.9700
O3—C5	1.437 (5)	C32—H32B	0.9700
O3—H3C	0.8198	C34—C44	1.532 (4)
O4—C22	1.254 (3)	C34—C36	1.536 (4)
O5—C22	1.254 (3)	C34—C37	1.539 (4)
O6—C16	1.437 (4)	C34—C35	1.539 (4)
O6—H6C	0.8202	C35—C40	1.531 (4)
O7—C33	1.257 (4)	C35—H35A	0.9700
O8—C33	1.265 (3)	C35—H35B	0.9700
O9—C27	1.429 (4)	C36—C38	1.520 (4)
O9—H9B	0.8199	C36—H36A	0.9700
O10—C44	1.249 (4)	C36—H36B	0.9700
O11—C44	1.273 (4)	C37—C42	1.535 (4)
O12—C38	1.430 (4)	C37—H37A	0.9700
O12—H12A	0.8205	C37—H37B	0.9700
N1—C47	1.335 (4)	C38—C39	1.522 (4)
N1—C48	1.347 (4)	C38—C43	1.528 (4)
N2—C52	1.328 (4)	C39—C40	1.521 (5)
N2—C53	1.334 (4)	C39—H39A	0.9700
C1—C11	1.522 (4)	C39—H39B	0.9700
C1—C4	1.524 (4)	C40—C41	1.530 (5)
C1—C2	1.536 (4)	C40—H40	0.9800
C1—C3	1.539 (4)	C41—C42	1.537 (5)
C2—C7	1.530 (5)	C41—H41A	0.9700
C2—H2A	0.9700	C41—H41B	0.9700
C2—H2B	0.9700	C42—C43	1.532 (5)
C3—C5	1.514 (4)	C42—H42	0.9800
C3—H3A	0.9700	C43—H43A	0.9700
C3—H3B	0.9700	C43—H43B	0.9700
C4—C9	1.536 (4)	C45—C49	1.391 (4)
C4—H4A	0.9700	C45—C46	1.395 (4)
C4—H4B	0.9700	C45—C61^i^	1.484 (4)
C5—C10	1.521 (5)	C46—C47	1.373 (4)
C5—C6	1.528 (6)	C46—H46	0.9300
C6—C7	1.516 (6)	C47—H47	0.9300
C6—H6A	0.9700	C48—C49	1.369 (4)
C6—H6B	0.9700	C48—H48	0.9300
C7—C8	1.537 (6)	C49—H49	0.9300
C7—H7	0.9800	C50—C54	1.380 (4)
C8—C9	1.521 (5)	C50—C51	1.382 (5)
C8—H8A	0.9700	C50—C56^ii^	1.484 (4)
C8—H8B	0.9700	C51—C52	1.362 (5)
C9—C10	1.518 (5)	C51—H51	0.9300
C9—H9A	0.9800	C52—H52	0.9300
C10—H10A	0.9700	C53—C54	1.377 (4)
C10—H10B	0.9700	C53—H53	0.9300
C12—C22	1.527 (4)	C54—H54	0.9300
C12—C14	1.536 (4)	O2W—H2WA	0.8499
C12—C13	1.537 (4)	O2W—H2WB	0.8500
C12—C15	1.543 (4)	O3W—H3WA	0.8501
C13—C16	1.527 (4)	O3W—H3WB	0.8500
C13—H13A	0.9700	O4W—H4WA	0.8444
C13—H13B	0.9700	O4W—H4WB	0.8213
C14—C18	1.529 (4)	O5W—H5WA	0.8500
C14—H14A	0.9700	O5W—H5WB	0.8494
C14—H14B	0.9700	O6W—H6WA	0.8499
C15—C20	1.538 (4)	O6W—H6WB	0.8502
C15—H15A	0.9700	O7W—H7WA	0.8500
C15—H15B	0.9700	O7W—H7WB	0.8500
C16—C17	1.508 (5)	O8W—H8WA	0.8293
C16—C21	1.519 (5)	O8W—H8WB	0.8540
C17—C18	1.516 (5)	C62—C63	1.376 (4)
C17—H17A	0.9700	C62—C61	1.389 (4)
C17—H17B	0.9700	C62—H62	0.9300
C18—C19	1.535 (5)	C65—C61	1.373 (4)
C18—H18A	0.9800	C65—C64	1.380 (4)
C19—C20	1.522 (5)	C65—H65	0.9300
C19—H19A	0.9700	N4—C63	1.334 (4)
C19—H19B	0.9700	N4—C64	1.338 (4)
C20—C21	1.505 (5)	C63—H63	0.9300
C20—H20	0.9800	C64—H64	0.9300
C21—H21A	0.9700	C61—C45^iii^	1.484 (4)
C21—H21B	0.9700	C56—C57	1.390 (4)
C23—C33	1.527 (4)	C56—C60	1.394 (4)
C23—C26	1.532 (4)	C56—C50^iv^	1.484 (4)
C23—C25	1.533 (4)	C57—C58	1.374 (4)
C23—C24	1.539 (4)	C57—H57	0.9300
C24—C29	1.531 (5)	C58—N3	1.343 (4)
C24—H24A	0.9700	C58—H58	0.9300
C24—H24B	0.9700	C59—N3	1.323 (4)
C25—C31	1.543 (5)	C59—C60	1.381 (4)
C25—H25A	0.9700	C59—H59	0.9300
C25—H25B	0.9700	C60—H60	0.9300
			
O7—Co1—O11	175.18 (9)	H24A—C24—H24B	108.2
O7—Co1—O5	88.17 (8)	C23—C25—C31	109.0 (3)
O11—Co1—O5	88.96 (8)	C23—C25—H25A	109.9
O7—Co1—N1	88.31 (9)	C31—C25—H25A	109.9
O11—Co1—N1	87.64 (9)	C23—C25—H25B	109.9
O5—Co1—N1	86.20 (9)	C31—C25—H25B	109.9
O7—Co1—O1W	95.00 (8)	H25A—C25—H25B	108.3
O11—Co1—O1W	88.95 (8)	C27—C26—C23	110.8 (3)
O5—Co1—O1W	91.80 (7)	C27—C26—H26A	109.5
N1—Co1—O1W	176.08 (9)	C23—C26—H26A	109.5
O7—Co1—N4	91.22 (9)	C27—C26—H26B	109.5
O11—Co1—N4	91.61 (9)	C23—C26—H26B	109.5
O5—Co1—N4	179.18 (9)	H26A—C26—H26B	108.1
N1—Co1—N4	93.23 (9)	O9—C27—C28	107.7 (3)
O1W—Co1—N4	88.79 (8)	O9—C27—C26	110.7 (3)
O4—Co2—O2	176.12 (8)	C28—C27—C26	109.4 (3)
O4—Co2—O8	92.80 (8)	O9—C27—C32	110.4 (3)
O2—Co2—O8	90.39 (8)	C28—C27—C32	109.4 (3)
O4—Co2—N2	90.03 (9)	C26—C27—C32	109.2 (3)
O2—Co2—N2	87.82 (9)	C29—C28—C27	109.8 (3)
O8—Co2—N2	89.16 (9)	C29—C28—H28A	109.7
O4—Co2—O1W	93.78 (8)	C27—C28—H28A	109.7
O2—Co2—O1W	88.46 (8)	C29—C28—H28B	109.7
O8—Co2—O1W	89.30 (7)	C27—C28—H28B	109.7
N2—Co2—O1W	175.96 (8)	H28A—C28—H28B	108.2
O4—Co2—N3	90.30 (9)	C28—C29—C30	109.9 (3)
O2—Co2—N3	86.62 (9)	C28—C29—C24	110.2 (3)
O8—Co2—N3	175.89 (9)	C30—C29—C24	109.2 (3)
N2—Co2—N3	93.55 (9)	C28—C29—H29	109.2
O1W—Co2—N3	87.79 (8)	C30—C29—H29	109.2
Co1—O1W—Co2	111.87 (8)	C24—C29—H29	109.2
Co1—O1W—H1WA	126.0	C31—C30—C29	109.1 (3)
Co2—O1W—H1WA	96.8	C31—C30—H30A	109.9
Co1—O1W—H1WB	100.9	C29—C30—H30A	109.9
Co2—O1W—H1WB	130.0	C31—C30—H30B	109.9
H1WA—O1W—H1WB	92.9	C29—C30—H30B	109.9
C11—O2—Co2	132.6 (2)	H30A—C30—H30B	108.3
C5—O3—H3C	109.2	C30—C31—C32	110.1 (3)
C22—O4—Co2	141.37 (19)	C30—C31—C25	110.2 (3)
C22—O5—Co1	127.35 (19)	C32—C31—C25	109.2 (3)
C16—O6—H6C	109.6	C30—C31—H31	109.1
C33—O7—Co1	138.71 (19)	C32—C31—H31	109.1
C33—O8—Co2	126.29 (18)	C25—C31—H31	109.1
C27—O9—H9B	109.5	C27—C32—C31	108.9 (3)
C44—O11—Co1	132.62 (19)	C27—C32—H32A	109.9
C38—O12—H12A	109.6	C31—C32—H32A	109.9
C47—N1—C48	116.7 (2)	C27—C32—H32B	109.9
C47—N1—Co1	122.9 (2)	C31—C32—H32B	109.9
C48—N1—Co1	119.55 (19)	H32A—C32—H32B	108.3
C52—N2—C53	116.1 (3)	O7—C33—O8	124.6 (3)
C52—N2—Co2	123.0 (2)	O7—C33—C23	117.2 (2)
C53—N2—Co2	120.6 (2)	O8—C33—C23	118.1 (3)
C11—C1—C4	111.7 (2)	C44—C34—C36	111.5 (2)
C11—C1—C2	112.0 (2)	C44—C34—C37	112.1 (2)
C4—C1—C2	109.2 (3)	C36—C34—C37	108.6 (2)
C11—C1—C3	106.4 (2)	C44—C34—C35	106.7 (2)
C4—C1—C3	108.4 (2)	C36—C34—C35	108.6 (2)
C2—C1—C3	109.0 (3)	C37—C34—C35	109.2 (2)
C7—C2—C1	109.5 (3)	C40—C35—C34	109.6 (3)
C7—C2—H2A	109.8	C40—C35—H35A	109.8
C1—C2—H2A	109.8	C34—C35—H35A	109.8
C7—C2—H2B	109.8	C40—C35—H35B	109.8
C1—C2—H2B	109.8	C34—C35—H35B	109.8
H2A—C2—H2B	108.2	H35A—C35—H35B	108.2
C5—C3—C1	110.7 (3)	C38—C36—C34	110.9 (2)
C5—C3—H3A	109.5	C38—C36—H36A	109.5
C1—C3—H3A	109.5	C34—C36—H36A	109.5
C5—C3—H3B	109.5	C38—C36—H36B	109.5
C1—C3—H3B	109.5	C34—C36—H36B	109.5
H3A—C3—H3B	108.1	H36A—C36—H36B	108.1
C1—C4—C9	109.7 (2)	C42—C37—C34	109.6 (2)
C1—C4—H4A	109.7	C42—C37—H37A	109.7
C9—C4—H4A	109.7	C34—C37—H37A	109.7
C1—C4—H4B	109.7	C42—C37—H37B	109.7
C9—C4—H4B	109.7	C34—C37—H37B	109.7
H4A—C4—H4B	108.2	H37A—C37—H37B	108.2
O3—C5—C3	110.2 (3)	O12—C38—C36	109.8 (2)
O3—C5—C10	107.1 (3)	O12—C38—C39	107.8 (2)
C3—C5—C10	109.2 (3)	C36—C38—C39	109.0 (2)
O3—C5—C6	111.4 (3)	O12—C38—C43	111.3 (2)
C3—C5—C6	109.5 (3)	C36—C38—C43	109.5 (2)
C10—C5—C6	109.4 (3)	C39—C38—C43	109.4 (3)
C7—C6—C5	109.6 (3)	C40—C39—C38	109.6 (2)
C7—C6—H6A	109.7	C40—C39—H39A	109.7
C5—C6—H6A	109.7	C38—C39—H39A	109.7
C7—C6—H6B	109.7	C40—C39—H39B	109.7
C5—C6—H6B	109.7	C38—C39—H39B	109.7
H6A—C6—H6B	108.2	H39A—C39—H39B	108.2
C6—C7—C2	109.8 (3)	C39—C40—C41	110.1 (3)
C6—C7—C8	109.7 (3)	C39—C40—C35	109.8 (3)
C2—C7—C8	109.3 (3)	C41—C40—C35	109.3 (3)
C6—C7—H7	109.4	C39—C40—H40	109.2
C2—C7—H7	109.4	C41—C40—H40	109.2
C8—C7—H7	109.4	C35—C40—H40	109.2
C9—C8—C7	109.1 (3)	C40—C41—C42	109.2 (3)
C9—C8—H8A	109.9	C40—C41—H41A	109.8
C7—C8—H8A	109.9	C42—C41—H41A	109.8
C9—C8—H8B	109.9	C40—C41—H41B	109.8
C7—C8—H8B	109.9	C42—C41—H41B	109.8
H8A—C8—H8B	108.3	H41A—C41—H41B	108.3
C10—C9—C8	109.7 (3)	C43—C42—C37	109.7 (3)
C10—C9—C4	110.3 (3)	C43—C42—C41	108.7 (3)
C8—C9—C4	109.0 (3)	C37—C42—C41	109.7 (3)
C10—C9—H9A	109.3	C43—C42—H42	109.6
C8—C9—H9A	109.3	C37—C42—H42	109.6
C4—C9—H9A	109.3	C41—C42—H42	109.6
C9—C10—C5	109.5 (3)	C38—C43—C42	109.9 (3)
C9—C10—H10A	109.8	C38—C43—H43A	109.7
C5—C10—H10A	109.8	C42—C43—H43A	109.7
C9—C10—H10B	109.8	C38—C43—H43B	109.7
C5—C10—H10B	109.8	C42—C43—H43B	109.7
H10A—C10—H10B	108.2	H43A—C43—H43B	108.2
O2—C11—O1	123.6 (3)	O10—C44—O11	123.5 (3)
O2—C11—C1	118.7 (3)	O10—C44—C34	120.5 (3)
O1—C11—C1	117.7 (3)	O11—C44—C34	115.9 (2)
C22—C12—C14	107.4 (2)	C49—C45—C46	116.0 (3)
C22—C12—C13	110.2 (2)	C49—C45—C61^i^	122.2 (3)
C14—C12—C13	108.4 (2)	C46—C45—C61^i^	121.8 (3)
C22—C12—C15	114.0 (2)	C47—C46—C45	120.1 (3)
C14—C12—C15	108.4 (3)	C47—C46—H46	119.9
C13—C12—C15	108.4 (3)	C45—C46—H46	119.9
C16—C13—C12	110.5 (3)	N1—C47—C46	123.5 (3)
C16—C13—H13A	109.5	N1—C47—H47	118.3
C12—C13—H13A	109.5	C46—C47—H47	118.3
C16—C13—H13B	109.5	N1—C48—C49	123.1 (3)
C12—C13—H13B	109.5	N1—C48—H48	118.4
H13A—C13—H13B	108.1	C49—C48—H48	118.4
C18—C14—C12	110.5 (3)	C48—C49—C45	120.5 (3)
C18—C14—H14A	109.5	C48—C49—H49	119.8
C12—C14—H14A	109.5	C45—C49—H49	119.8
C18—C14—H14B	109.5	C54—C50—C51	115.9 (3)
C12—C14—H14B	109.5	C54—C50—C56^ii^	121.7 (3)
H14A—C14—H14B	108.1	C51—C50—C56^ii^	122.4 (3)
C20—C15—C12	109.5 (2)	C52—C51—C50	120.8 (3)
C20—C15—H15A	109.8	C52—C51—H51	119.6
C12—C15—H15A	109.8	C50—C51—H51	119.6
C20—C15—H15B	109.8	N2—C52—C51	123.6 (3)
C12—C15—H15B	109.8	N2—C52—H52	118.2
H15A—C15—H15B	108.2	C51—C52—H52	118.2
O6—C16—C17	107.3 (3)	N2—C53—C54	123.8 (3)
O6—C16—C21	111.7 (3)	N2—C53—H53	118.1
C17—C16—C21	110.0 (3)	C54—C53—H53	118.1
O6—C16—C13	109.3 (3)	C53—C54—C50	119.7 (3)
C17—C16—C13	109.6 (3)	C53—C54—H54	120.1
C21—C16—C13	108.9 (3)	C50—C54—H54	120.1
C16—C17—C18	109.8 (3)	H2WA—O2W—H2WB	102.9
C16—C17—H17A	109.7	H3WA—O3W—H3WB	128.8
C18—C17—H17A	109.7	H4WA—O4W—H4WB	102.0
C16—C17—H17B	109.7	H5WA—O5W—H5WB	90.3
C18—C17—H17B	109.7	H6WA—O6W—H6WB	118.6
H17A—C17—H17B	108.2	H7WA—O7W—H7WB	105.6
C17—C18—C14	109.6 (3)	H8WA—O8W—H8WB	102.9
C17—C18—C19	110.0 (3)	C63—C62—C61	120.0 (3)
C14—C18—C19	108.5 (3)	C63—C62—H62	120.0
C17—C18—H18A	109.5	C61—C62—H62	120.0
C14—C18—H18A	109.5	C61—C65—C64	120.6 (3)
C19—C18—H18A	109.5	C61—C65—H65	119.7
C20—C19—C18	108.9 (3)	C64—C65—H65	119.7
C20—C19—H19A	109.9	C63—N4—C64	115.9 (2)
C18—C19—H19A	109.9	C63—N4—Co1	120.95 (19)
C20—C19—H19B	109.9	C64—N4—Co1	123.0 (2)
C18—C19—H19B	109.9	N4—C63—C62	123.9 (3)
H19A—C19—H19B	108.3	N4—C63—H63	118.0
C21—C20—C19	110.5 (3)	C62—C63—H63	118.0
C21—C20—C15	109.9 (3)	N4—C64—C65	123.5 (3)
C19—C20—C15	109.1 (3)	N4—C64—H64	118.2
C21—C20—H20	109.1	C65—C64—H64	118.2
C19—C20—H20	109.1	C65—C61—C62	116.1 (3)
C15—C20—H20	109.1	C65—C61—C45^iii^	122.2 (3)
C20—C21—C16	109.8 (3)	C62—C61—C45^iii^	121.7 (3)
C20—C21—H21A	109.7	C57—C56—C60	116.2 (3)
C16—C21—H21A	109.7	C57—C56—C50^iv^	121.5 (3)
C20—C21—H21B	109.7	C60—C56—C50^iv^	122.4 (3)
C16—C21—H21B	109.7	C58—C57—C56	120.2 (3)
H21A—C21—H21B	108.2	C58—C57—H57	119.9
O5—C22—O4	124.9 (3)	C56—C57—H57	119.9
O5—C22—C12	119.8 (3)	N3—C58—C57	123.3 (3)
O4—C22—C12	115.2 (2)	N3—C58—H58	118.3
C33—C23—C26	109.1 (2)	C57—C58—H58	118.3
C33—C23—C25	110.8 (2)	N3—C59—C60	124.0 (3)
C26—C23—C25	109.0 (3)	N3—C59—H59	118.0
C33—C23—C24	110.5 (2)	C60—C59—H59	118.0
C26—C23—C24	108.1 (2)	C59—N3—C58	116.6 (3)
C25—C23—C24	109.3 (3)	C59—N3—Co2	122.4 (2)
C29—C24—C23	109.5 (3)	C58—N3—Co2	119.9 (2)
C29—C24—H24A	109.8	C59—C60—C56	119.6 (3)
C23—C24—H24A	109.8	C59—C60—H60	120.2
C29—C24—H24B	109.8	C56—C60—H60	120.2
C23—C24—H24B	109.8		
			
O7—Co1—O1W—Co2	−35.35 (10)	C26—C23—C25—C31	−59.5 (3)
O11—Co1—O1W—Co2	141.90 (10)	C24—C23—C25—C31	58.5 (4)
O5—Co1—O1W—Co2	52.97 (10)	C33—C23—C26—C27	−179.6 (2)
N4—Co1—O1W—Co2	−126.47 (10)	C25—C23—C26—C27	59.3 (3)
O4—Co2—O1W—Co1	−38.24 (10)	C24—C23—C26—C27	−59.4 (3)
O2—Co2—O1W—Co1	144.93 (10)	C23—C26—C27—O9	178.5 (2)
O8—Co2—O1W—Co1	54.52 (10)	C23—C26—C27—C28	60.0 (3)
N3—Co2—O1W—Co1	−128.39 (10)	C23—C26—C27—C32	−59.8 (4)
O8—Co2—O2—C11	92.3 (3)	O9—C27—C28—C29	−179.7 (3)
N2—Co2—O2—C11	−178.5 (3)	C26—C27—C28—C29	−59.4 (4)
O1W—Co2—O2—C11	3.0 (3)	C32—C27—C28—C29	60.2 (4)
N3—Co2—O2—C11	−84.9 (3)	C27—C28—C29—C30	−60.4 (4)
O8—Co2—O4—C22	−84.9 (3)	C27—C28—C29—C24	60.0 (4)
N2—Co2—O4—C22	−174.1 (3)	C23—C24—C29—C28	−60.0 (4)
O1W—Co2—O4—C22	4.6 (3)	C23—C24—C29—C30	60.9 (4)
N3—Co2—O4—C22	92.4 (3)	C28—C29—C30—C31	59.7 (4)
O7—Co1—O5—C22	41.3 (2)	C24—C29—C30—C31	−61.3 (4)
O11—Co1—O5—C22	−142.5 (2)	C29—C30—C31—C32	−59.5 (4)
N1—Co1—O5—C22	129.8 (2)	C29—C30—C31—C25	60.9 (4)
O1W—Co1—O5—C22	−53.6 (2)	C23—C25—C31—C30	−59.7 (4)
O5—Co1—O7—C33	−96.2 (3)	C23—C25—C31—C32	61.4 (4)
N1—Co1—O7—C33	177.5 (3)	O9—C27—C32—C31	−177.7 (3)
O1W—Co1—O7—C33	−4.6 (3)	C28—C27—C32—C31	−59.3 (4)
N4—Co1—O7—C33	84.3 (3)	C26—C27—C32—C31	60.4 (4)
O4—Co2—O8—C33	32.3 (2)	C30—C31—C32—C27	59.6 (4)
O2—Co2—O8—C33	−149.9 (2)	C25—C31—C32—C27	−61.6 (4)
N2—Co2—O8—C33	122.3 (2)	Co1—O7—C33—O8	10.3 (5)
O1W—Co2—O8—C33	−61.4 (2)	Co1—O7—C33—C23	−166.5 (2)
O5—Co1—O11—C44	101.9 (3)	Co2—O8—C33—O7	32.6 (4)
N1—Co1—O11—C44	−171.9 (3)	Co2—O8—C33—C23	−150.6 (2)
O1W—Co1—O11—C44	10.0 (3)	C26—C23—C33—O7	107.6 (3)
N4—Co1—O11—C44	−78.7 (3)	C25—C23—C33—O7	−132.5 (3)
O7—Co1—N1—C47	−146.9 (2)	C24—C23—C33—O7	−11.2 (4)
O11—Co1—N1—C47	35.7 (2)	C26—C23—C33—O8	−69.4 (3)
O5—Co1—N1—C47	124.9 (2)	C25—C23—C33—O8	50.5 (4)
N4—Co1—N1—C47	−55.7 (2)	C24—C23—C33—O8	171.8 (3)
O7—Co1—N1—C48	44.2 (2)	C44—C34—C35—C40	178.7 (2)
O11—Co1—N1—C48	−133.2 (2)	C36—C34—C35—C40	58.4 (3)
O5—Co1—N1—C48	−44.1 (2)	C37—C34—C35—C40	−59.9 (3)
N4—Co1—N1—C48	135.3 (2)	C44—C34—C36—C38	−176.4 (2)
O4—Co2—N2—C52	151.4 (3)	C37—C34—C36—C38	59.6 (3)
O2—Co2—N2—C52	−31.8 (3)	C35—C34—C36—C38	−59.1 (3)
O8—Co2—N2—C52	58.6 (3)	C44—C34—C37—C42	177.1 (2)
N3—Co2—N2—C52	−118.3 (3)	C36—C34—C37—C42	−59.2 (3)
O4—Co2—N2—C53	−21.5 (2)	C35—C34—C37—C42	59.1 (3)
O2—Co2—N2—C53	155.3 (2)	C34—C36—C38—O12	177.9 (2)
O8—Co2—N2—C53	−114.3 (2)	C34—C36—C38—C39	60.0 (3)
N3—Co2—N2—C53	68.8 (2)	C34—C36—C38—C43	−59.6 (3)
C11—C1—C2—C7	176.0 (3)	O12—C38—C39—C40	−179.3 (2)
C4—C1—C2—C7	−59.7 (4)	C36—C38—C39—C40	−60.2 (3)
C3—C1—C2—C7	58.5 (4)	C43—C38—C39—C40	59.5 (3)
C11—C1—C3—C5	−179.6 (2)	C38—C39—C40—C41	−59.7 (3)
C4—C1—C3—C5	60.1 (3)	C38—C39—C40—C35	60.7 (3)
C2—C1—C3—C5	−58.6 (3)	C34—C35—C40—C39	−60.0 (3)
C11—C1—C4—C9	−175.5 (3)	C34—C35—C40—C41	61.0 (3)
C2—C1—C4—C9	60.1 (3)	C39—C40—C41—C42	59.9 (3)
C3—C1—C4—C9	−58.5 (3)	C35—C40—C41—C42	−60.8 (4)
C1—C3—C5—O3	−178.0 (3)	C34—C37—C42—C43	59.9 (3)
C1—C3—C5—C10	−60.6 (4)	C34—C37—C42—C41	−59.5 (3)
C1—C3—C5—C6	59.1 (4)	C40—C41—C42—C43	−59.8 (4)
O3—C5—C6—C7	178.0 (3)	C40—C41—C42—C37	60.2 (3)
C3—C5—C6—C7	−59.9 (4)	O12—C38—C43—C42	−179.4 (2)
C10—C5—C6—C7	59.8 (4)	C36—C38—C43—C42	59.0 (3)
C5—C6—C7—C2	60.6 (4)	C39—C38—C43—C42	−60.4 (3)
C5—C6—C7—C8	−59.4 (4)	C37—C42—C43—C38	−59.5 (3)
C1—C2—C7—C6	−60.3 (4)	C41—C42—C43—C38	60.5 (3)
C1—C2—C7—C8	60.1 (4)	Co1—O11—C44—O10	−16.1 (4)
C6—C7—C8—C9	59.5 (4)	Co1—O11—C44—C34	160.18 (19)
C2—C7—C8—C9	−60.9 (4)	C36—C34—C44—O10	−143.6 (3)
C7—C8—C9—C10	−59.9 (4)	C37—C34—C44—O10	−21.6 (4)
C7—C8—C9—C4	61.0 (4)	C35—C34—C44—O10	97.9 (3)
C1—C4—C9—C10	59.5 (3)	C36—C34—C44—O11	40.0 (3)
C1—C4—C9—C8	−61.1 (4)	C37—C34—C44—O11	162.0 (3)
C8—C9—C10—C5	60.6 (4)	C35—C34—C44—O11	−78.4 (3)
C4—C9—C10—C5	−59.4 (4)	C49—C45—C46—C47	−2.3 (4)
O3—C5—C10—C9	179.0 (3)	C61^i^—C45—C46—C47	177.5 (3)
C3—C5—C10—C9	59.7 (4)	C48—N1—C47—C46	2.1 (5)
C6—C5—C10—C9	−60.1 (4)	Co1—N1—C47—C46	−167.1 (2)
Co2—O2—C11—O1	−22.4 (5)	C45—C46—C47—N1	0.5 (5)
Co2—O2—C11—C1	155.8 (2)	C47—N1—C48—C49	−3.0 (4)
C4—C1—C11—O2	12.5 (4)	Co1—N1—C48—C49	166.6 (2)
C2—C1—C11—O2	135.3 (3)	N1—C48—C49—C45	1.2 (5)
C3—C1—C11—O2	−105.6 (3)	C46—C45—C49—C48	1.5 (4)
C4—C1—C11—O1	−169.2 (3)	C61^i^—C45—C49—C48	−178.3 (3)
C2—C1—C11—O1	−46.4 (4)	C54—C50—C51—C52	−3.7 (6)
C3—C1—C11—O1	72.6 (3)	C56^ii^—C50—C51—C52	176.5 (3)
C22—C12—C13—C16	175.4 (2)	C53—N2—C52—C51	0.5 (6)
C14—C12—C13—C16	58.1 (3)	Co2—N2—C52—C51	−172.7 (3)
C15—C12—C13—C16	−59.4 (3)	C50—C51—C52—N2	2.0 (7)
C22—C12—C14—C18	−176.9 (3)	C52—N2—C53—C54	−1.0 (5)
C13—C12—C14—C18	−57.9 (3)	Co2—N2—C53—C54	172.4 (3)
C15—C12—C14—C18	59.6 (3)	N2—C53—C54—C50	−1.0 (5)
C22—C12—C15—C20	−178.6 (3)	C51—C50—C54—C53	3.2 (5)
C14—C12—C15—C20	−59.2 (3)	C56^ii^—C50—C54—C53	−177.0 (3)
C13—C12—C15—C20	58.3 (3)	O7—Co1—N4—C63	31.1 (2)
C12—C13—C16—O6	−177.2 (3)	O11—Co1—N4—C63	−145.0 (2)
C12—C13—C16—C17	−59.9 (3)	N1—Co1—N4—C63	−57.3 (2)
C12—C13—C16—C21	60.4 (4)	O1W—Co1—N4—C63	126.1 (2)
O6—C16—C17—C18	178.9 (3)	O7—Co1—N4—C64	−143.1 (2)
C21—C16—C17—C18	−59.4 (4)	O11—Co1—N4—C64	40.8 (2)
C13—C16—C17—C18	60.3 (4)	N1—Co1—N4—C64	128.5 (2)
C16—C17—C18—C14	−60.1 (4)	O1W—Co1—N4—C64	−48.1 (2)
C16—C17—C18—C19	59.2 (4)	C64—N4—C63—C62	0.5 (5)
C12—C14—C18—C17	59.4 (4)	Co1—N4—C63—C62	−174.1 (3)
C12—C14—C18—C19	−60.8 (4)	C61—C62—C63—N4	−0.9 (5)
C17—C18—C19—C20	−58.4 (4)	C63—N4—C64—C65	0.2 (5)
C14—C18—C19—C20	61.5 (4)	Co1—N4—C64—C65	174.7 (3)
C18—C19—C20—C21	58.8 (4)	C61—C65—C64—N4	−0.5 (5)
C18—C19—C20—C15	−62.2 (4)	C64—C65—C61—C62	0.1 (5)
C12—C15—C20—C21	−60.0 (4)	C64—C65—C61—C45^iii^	−179.5 (3)
C12—C15—C20—C19	61.3 (4)	C63—C62—C61—C65	0.5 (5)
C19—C20—C21—C16	−59.5 (4)	C63—C62—C61—C45^iii^	−179.8 (3)
C15—C20—C21—C16	61.0 (4)	C60—C56—C57—C58	0.9 (4)
O6—C16—C21—C20	178.4 (3)	C50^iv^—C56—C57—C58	179.6 (3)
C17—C16—C21—C20	59.4 (4)	C56—C57—C58—N3	−0.2 (5)
C13—C16—C21—C20	−60.7 (4)	C60—C59—N3—C58	2.5 (5)
Co1—O5—C22—O4	26.9 (4)	C60—C59—N3—Co2	−165.5 (2)
Co1—O5—C22—C12	−153.9 (2)	C57—C58—N3—C59	−1.5 (4)
Co2—O4—C22—O5	4.4 (5)	C57—C58—N3—Co2	166.8 (2)
Co2—O4—C22—C12	−174.7 (2)	O4—Co2—N3—C59	−55.8 (2)
C14—C12—C22—O5	−112.9 (3)	O2—Co2—N3—C59	126.5 (2)
C13—C12—C22—O5	129.1 (3)	N2—Co2—N3—C59	−145.9 (2)
C15—C12—C22—O5	7.1 (4)	O1W—Co2—N3—C59	37.9 (2)
C14—C12—C22—O4	66.3 (3)	O4—Co2—N3—C58	136.6 (2)
C13—C12—C22—O4	−51.6 (3)	O2—Co2—N3—C58	−41.1 (2)
C15—C12—C22—O4	−173.7 (3)	N2—Co2—N3—C58	46.5 (2)
C33—C23—C24—C29	178.0 (3)	O1W—Co2—N3—C58	−129.7 (2)
C26—C23—C24—C29	58.7 (4)	N3—C59—C60—C56	−1.8 (5)
C25—C23—C24—C29	−59.8 (4)	C57—C56—C60—C59	0.0 (4)
C33—C23—C25—C31	−179.5 (3)	C50^iv^—C56—C60—C59	−178.6 (3)

Symmetry codes: (i) *x*, −*y*−2, *z*+1/2; (ii) *x*+1, −*y*−1, *z*+1/2; (iii) *x*, −*y*−2, *z*−1/2; (iv) *x*−1, −*y*−1, *z*−1/2.

### Hydrogen-bond geometry (Å, °)

**Table:**

*D*—H···*A*	*D*—H	H···*A*	*D*···*A*	*D*—H···*A*
O1W—H1WA···O1	0.85	1.78	2.623 (3)	168
O1W—H1WA···O2	0.85	2.56	2.968 (3)	111
O1W—H1WB···O10	0.95	1.69	2.623 (3)	164
O3W—H3WA···O5W	0.85	2.01	2.844 (5)	166
O3W—H3WB···O8W	0.85	2.12	2.973 (5)	179
O12—H12A···O4W	0.82	2.03	2.836 (4)	170
O6W—H6WA···O2W	0.85	2.08	2.865 (8)	154
O6—H6C···O8W	0.82	2.10	2.923 (4)	180
O6W—H6WB···O7W	0.85	2.08	2.920 (7)	167
O7W—H7WB···O5	0.85	2.07	2.794 (3)	143
O7W—H7WA···O11	0.85	2.12	2.908 (3)	155
O3—H3C···O6^v^	0.82	2.07	2.884 (4)	176
O9—H9B···O7W^vi^	0.82	2.04	2.864 (4)	178
O2W—H2WA···O3^vii^	0.85	2.03	2.881 (6)	179
O4W—H4WA···O8^viii^	0.84	2.10	2.943 (3)	176
O4W—H4WB···O1^viii^	0.82	2.09	2.843 (4)	152
O5W—H5WA···O12^ix^	0.85	2.00	2.849 (4)	180
O5W—H5WB···O9^i^	0.85	2.11	2.927 (4)	163
O8W—H8WA···O1^x^	0.83	2.08	2.882 (4)	163
O8W—H8WB···O10^x^	0.85	2.01	2.857 (4)	173
O2W—H2WA···O3^vii^	0.85	2.03	2.881 (6)	179

Symmetry codes: (v) *x*−1, *y*, *z*−1; (vi) *x*+1, *y*, *z*; (vii) *x*, −*y*−1, *z*+1/2; (viii) *x*−1, *y*, *z*; (ix) *x*+1, −*y*−2, *z*+1/2; (i) *x*, −*y*−2, *z*+1/2; (x) *x*+1, *y*, *z*+1.
